# Exploring the role of gut microbiota in colorectal liver metastasis through the gut-liver axis

**DOI:** 10.3389/fcell.2025.1563184

**Published:** 2025-03-13

**Authors:** Qiu-Luo Liu, Huijie Zhou, Ziqiang Wang, Yan Chen

**Affiliations:** ^1^ Colorectal Cancer Center, Department of General Surgery, West China Hospital, Sichuan University, Chengdu, China; ^2^ Institute of Digestive Surgery, Institute of General Surgery, West China Hospital, Sichuan University, Chengdu, China; ^3^ State Key Laboratory of Biotherapy and Cancer Center, West China Hospital, Sichuan University, Chengdu, Sichuan, China; ^4^ Health Management Center, General Practice Center, West China Hospital, Sichuan University, Chengdu, China; ^5^ Department of Gastrointestinal Surgery, Sichuan Provincial People’s Hospital, University of Electronic Science and Technology of China, Chengdu, China

**Keywords:** colorectal cancer liver metastasis, gut microbiota, metabolites, gut-liver axis, tumor immune microenvironment

## Abstract

Colorectal liver metastasis (CRLM) represents a major therapeutic challenge in colorectal cancer (CRC), with complex interactions between the gut microbiota and the liver tumor microenvironment (TME) playing a crucial role in disease progression via the gut-liver axis. The gut barrier serves as a gatekeeper, regulating microbial translocation, which influences liver colonization and metastasis. Through the gut-liver axis, the microbiota actively shapes the TME, where specific microbial species and their metabolites exert dual roles in immune modulation. The immunologically “cold” nature of the liver, combined with the influence of the gut microbiota on liver immunity, complicates effective immunotherapy. However, microbiota-targeted interventions present promising strategies to enhance immunotherapy outcomes by modulating the gut-liver axis. Overall, this review highlights the emerging evidence on the role of the gut microbiota in CRLM and provides insights into the molecular mechanisms driving the dynamic interactions within the gut-liver axis.

## 1 Introduction

Colorectal cancer (CRC) is the third most prevalent malignancy globally, accounting for approximately 10% of all cancer cases, and remains the second leading cause of cancer-related mortality worldwide (Siegel et al., 2024). Among metastatic patterns in CRC, colorectal liver metastasis (CRLM) is the most common and represents the primary cause of death in CRC patients (Ciraci et al., 2024). Approximately 30%–50% of CRC patients develop CRLM during the disease course, underscoring its clinical significance (Siegel et al., 2023). Notably, the incidence of metastatic liver cancer surpasses that of primary liver cancer by 18–40 times, with CRC being the most frequent primary source of liver metastases (Tsilimigras et al., 2021).

The tendency of CRC to preferentially metastasize to the liver can be explained by the liver’s unique blood supply, with 75% of blood flow through the portal vein from the colon and rectum, provides a direct route for CRC cells to reach the liver (Chow and Chok, 2019; Vidal-Vanaclocha, 2008). The hepatic sinusoids, where blood flow is slow and the vascular permeability is high, also create a favorable environment for tumor cell retention and colonization (Mielgo and Schmid, 2020; Poisson et al., 2017). However, it is important to recognize that metastasis is a systemic process, and the portal vein also carries a wide range of substances besides tumor cells.

The gut, where CRC originates, is home to a highly complex and dynamic microbiota, which plays a pivotal role in the development and progression of CRC. Dysbiosis, characterized by an imbalance in microbial composition, is a hallmark of CRC, fostering chronic inflammation and driving tumor progression. Specific microbial species, such as *Fusobacterium nucleatum* (Zepeda-Rivera et al., 2024; Wang and Fang, 2023; Yu et al., 2017), *Bacteroides fragilis* (Purcell et al., 2017; Chung et al., 2018a; Boleij et al., 2015), and *Escherichia coli* (Swidsinski et al., 1998; Bonnet et al., 2014; Prorok-Hamon et al., 2014), have been linked to enhanced tumor invasiveness through direct interactions with CRC cells and by modulating the immune microenvironment in a tumor-promoting direction (Tilg et al., 2018; Gao et al., 2022; Sears and Garrett, 2014). Moreover, microbial metabolites, such as short-chain fatty acids (SCFAs) and bile acids, can have dual roles, either protecting against or exacerbating tumorigenesis, depending on the broader environmental context (Cai et al., 2022; Cong et al., 2024; Ohigashi et al., 2013; Ocvirk and O'Keefe, 2021). These microbial products, along with other byproducts, travel to the liver via the portal vein, establishing a dynamic gut-liver connection, known as the gut-liver axis (Tilg et al., 2022).

Under normal conditions, the liver processes these molecules and maintains systemic homeostasis through bile acid secretion and immune responses (Pabst et al., 2023). However, CRC and dysbiosis can overwhelm these regulatory mechanisms, leading to a disrupted gut-liver axis (Albillos et al., 2020). This disruption facilitates the establishment of a pro-metastatic niche in the liver, marked by immune suppression and inflammation, creating a favorable environment for CRC cells to colonize and thrive. Therefore, this review explores the impact of the gut microbiota on CRLM through the gut-liver axis, focusing on the complex interplay between gut barrier, dysbiosis, microbial metabolites, and inflammation within the liver tumor microenvironment (TME). In addition, we discuss potential microbiota-targeted therapies, such as probiotics and prebiotics, which may offer new strategies to improve therapeutic outcomes in CRLM. Our goal is to provide a comprehensive perspective on CRLM and the gut-liver axis in the hope that bench discoveries can be effectively translated into clinical applications.

## 2 The gut barrier as the gatekeeper of the gut-liver axis

The gut barrier is a complex, multi-layered structure that extends from the mucus layer to epithelial tight junctions (TJs), host-microbe interactions, and the gut-vascular barrier (GVB) (Leshem et al., 2020; Rao et al., 2021). It serves as a protective defense, shielding the body from harmful substances such as bacteria and toxins. The GVB, as the key component, consisting of endothelial cells, pericytes, and enteric glia, specifically prevents bacteria and metabolites from translocating from the gut to the liver (Grander et al., 2020). While the intestinal barrier regulates gut permeability, the GVB offers an additional layer of defense at the gut-liver interface, underscoring the essential role of the gut barrier in maintaining gut-liver homeostasis (Spadoni et al., 2015). Disruption of the gut barrier can lead to liver diseases (Mouries et al., 2019).

**FIGURE 1:**
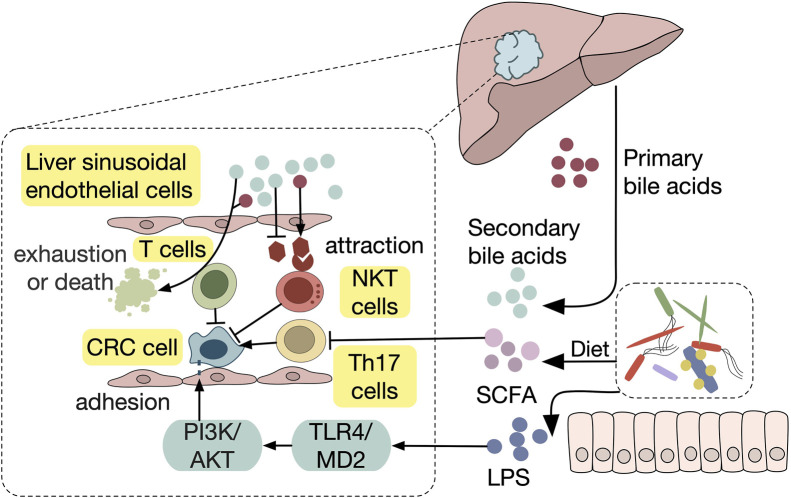
Impaired gut barrier enables CRLM The impaired gut barrier is a pivotal mediator of gut-liver axis dysfunction, facilitating CRLM. Detrimental factors such as operative trauma, chemotherapy, dysbiosis, antibiotics, and high-fat diets compromise gut epithelial integrity, resulting in increased permeability. Leaky epithelium and GVB allow microbial products, including bacteria and their metabolites, to translocate via the portal vein to the liver. This process triggers pre-metastatic niche formation in the liver, characterized by extracellular matrix (ECM) remodeling and chemoattractant production.

Increased intestinal permeability, commonly referred to as “leaky gut,” is a key factor in the development of liver diseases, especially through the gut-liver axis (Aburto and Cryan, 2024). Disruption of the GVB is closely linked to dysbiosis of the intestinal microbiome (Grander et al., 2018; Depommier et al., 2019). Changes in the composition of mucin and thickness of this layer, shaped by the microbiota, have been observed in pre-epithelial mucus layer in the colon of rats (Szentkuti et al., 1990). This barrier also can be compromised in patients undergoing treatment for CRC, where factors such as operative trauma, post-surgical complications, chemotherapy cytotoxicity, and antibiotics, can severely damage gut integrity (Yang et al., 2024; Schietroma et al., 2016; Gibiino et al., 2022). For instance, dysbiosis induced by a high-fat diet has been shown to result in intestinal barrier disruption, facilitating the entry of microbial components and metabolites into the bloodstream. This disruption is considered a precursor for the development of liver disorders including non-alcoholic fatty liver disease, steatohepatitis, and hepatocellular carcinoma (HCC) (Brescia and Rescigno, 2021). Furthermore, gut vascular impairment is linked to the formation of a “pre-metastatic niche” in the liver, where molecular and cellular changes at distant sites attract circulating tumor cells and support future metastatic growth (Peinado et al., 2017; Bertocchi et al., 2021) (Figure 1). These observations emphasize the crucial role of maintaining the physical integrity of the gut barrier in the gut-liver axis to prevent disease progression.

Disruption of the GVB plays a pivotal role in the formation of a pre-metastatic niche in liver metastasis of CRC (Murota and Jobin, 2021). Under normal conditions, the GVB acts as a critical protective layer that prevents bacteria and other large molecules from translocating across the intestinal epithelium into the bloodstream. However, when the GVB is compromised—such as during CRC or intestinal infection—bacteria can migrate from the gut into the liver (Ray, 2021). Notably, *E. coli*, particularly those expressing the Virf1 virulence factor, have been found to breach the GVB, triggering a cascade of immune and extracellular matrix changes in the liver that foster a pre-metastatic environment. This disruption not only facilitates bacterial dissemination to the liver but also creates favorable conditions for the seeding and growth of metastatic cancer cells. Elevated levels of PV-1, a marker for GVB disruption, in primary tumors have been linked to increased bacterial presence in liver metastases, further emphasizing the connection between GVB breakdown and CRC liver metastasis (Bertocchi et al., 2021). These findings highlight the crucial role of GVB integrity in microbial translocation via the gut-liver axis and emphasize its potential as a therapeutic target for preventing the formation of pre-metastatic niches in CRC liver metastasis.

While the GVB plays a critical role in maintaining microbial integrity and preventing the translocation of harmful bacteria, any disruption in this barrier can lead to severe complications. One such complication is an anastomotic leak, which occurs when the integrity of a surgical connection between two bowel segments is compromised (Figure 1). Similar to the breakdown of the GVB, an anastomotic leak allows bacterial dissemination and can lead to systemic infections, increasing the risk of CRLM (Mirnezami et al., 2011; Goto et al., 2017). Recent studies in mouse models revealed that anastomotic leak was associated with microbiota mediated systemic inflammation and development of CRLM (Hajjar et al., 2024; Hajjar et al., 2023). Specifically, the microbiota in patients with anastomotic leak showed diminished capacity to activate PPAR-γ, a receptor that plays a critical role in antineoplastic defense in the gut. Dietary interventions, such as inulin and 5-aminosalicylate (5-ASA), which activate PPAR-γ, were found to enhance gut barrier integrity, reduce the formation of anastomotic tumors, and prevent metastatic spread to the liver in mice (Hajjar et al., 2024). These findings underscore the importance of preventing anastomotic leak to improve oncological outcomes after CRC surgery and suggest that dietary modulation of the gut microbiota offers a novel strategy for promoting anastomotic healing and reducing CRLM.

## 3 Influence of the gut microbiota on CRLM via the gut-liver axis

The gut microbiota significantly influences the inflammatory environment in CRC, contributing to tumor initiation and progression. Dysbiosis, or an imbalance in the gut microbiome, has been linked to the promotion of colonic inflammation, a key factor in CRC initiation and progression. Various pathobionts, such as *F. nucleatum*, *B. fragilis*, and *Enterococcus faecalis*, have been implicated in this process. These microorganisms activate inflammatory pathways, including IL-17, NF-κB, and pattern recognition receptors, which not only exacerbate inflammation but also impair the gut barrier function (Kostic et al., 2013; Wu et al., 2009; Ruiz et al., 2005). In particular, *B. fragilis* has been shown to initiate an inflammatory cascade by disrupting the gut barrier, leading to the activation of TH17 cells and further propagation of the inflammatory response (Chung et al., 2018b). This ongoing inflammation is closely linked to the development of CRC, as it creates a tumor-promoting environment by altering immune TME.

The modulation of the gut immune microenvironment by microbiota also plays a significant role in promoting CRC local invasion and distant metastasis. Microbial metabolites, such as formate produced by *F. nucleatum*, have been shown to drive cancer cell invasion and metastatic spread by activating key signaling pathways, including the aryl hydrocarbon receptor, which enhances cancer stemness (Ternes et al., 2022). Furthermore, diet-induced dysbiosis, particularly from a high-fat diet, can exacerbate gut inflammation and barrier dysfunction, creating a favorable environment for both CRC initiation and metastasis. For instance, increased abundance of *Desulfovibrio* in high-fat diet models contributes to inflammation in both the colorectum and liver, highlighting the link between gut microbiota changes and CRC metastasis (Yu et al., 2022). Together, these observations highlight that the gut microenvironment plays a foundational role in the gut-liver axis, setting the stage for metastasis.

### 3.1 Microbiota translocation: The gut-liver axis as a pathway

The integrity of the gut barrier and the composition of the gut microbiota are intricately linked within the framework of the gut-liver axis, forming a dynamic system that regulates intestinal homeostasis and liver microenvironment. On the one hand, alterations in the gut microbiota—often manifesting as dysbiosis—can impair gut barrier function, exacerbating its permeability and fostering a cycle of microbial translocation. On the other hand, disruption of the gut barrier, through factors such as inflammation or infection, can create pathways for the translocation of gut microbiota into the liver, potentially enhancing metastatic dissemination and contributing to the formation of pre-metastatic niches in colorectal cancer.

**FIGURE 2:**
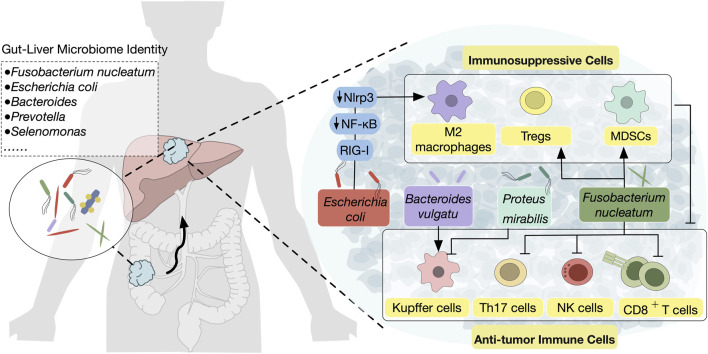
Microbiota translocation from the gut to the liver modulates the hepatic TME The complex interplay between gut microbiota and CRLM through the gut-liver axis are shown. Pathogenic bacteria such as *Fusobacterium nucleatum, Escherichia coli, and Proteus mirabilis* are translocated from the gut to the liver, where they contribute to tumor progression by inducing an immunosuppressive TME. Mechanisms include promoting the activation of MDSCs, Tregs, and M2 macrophages, which suppress anti-tumor immune populations such as NK cells, CD8^+^ T cells, and Kupffer cells. Conversely, certain bacterial species, such as *Bacteroides vulgatus*, can enhance anti-tumor immunity by increasing Kupffer cell activation.

The identity of microorganisms in primary and metastatic CRC suggests their translocation through the gut-liver axis. *Fusobacterium nucleatum* has been detected in 8%–13% of primary CRC in patient specimens (Nosho et al., 2016; Mima et al., 2015), with its abundance notably higher in CRC tissues from patients with metastasis (Chen et al., 2020). Notable, this bacterium is not only present in primary tumors but also in matched liver metastases, highlighting the stability of the microbiome between primary and metastatic sites (Bullman et al., 2017). Additionally, other associated species such as *Bacteroides*, *Selenomonas*, and *Prevotella* also remain consistent across both primary and metastatic tumors (Bullman et al., 2017) (Figure 2). These observations of microbiome stability from human samples provide direct evidence for the translocation of microbiome composition via the gut-liver axis, raising questions about if/how the gut microorganisms can directly influence its microenvironment.

### 3.2 Microbiota modulation: impact on the liver TME

The complex interplay between gut microbiota and the liver TME has garnered increasing attention, particularly regarding its role in modulating inflammatory responses and immune functions (Figure 2). For instance, a high-fat diet-induced microbiota imbalance, particularly with the abundance of *Desulfovibrio*, has been shown to promote gut barrier dysfunction and gut inflammation, creating a pro-inflammatory environment in the liver that favors CRC metastasis (Yu et al., 2022). Further investigation has revealed the involvement of specific bacteria in modulating immune responses. The balance of bacterial populations influences liver-resident Kupffer cells. Specifically, *Bacteroides vulgatus* has been linked to an increase in the proliferation of Kupffer cells, which subsequently reduces the incidence of CRLM. Conversely, bacteria such as *Proteus mirabilis* can lead to a reduction in Kupffer cells, promoting a more permissive environment for disseminating CRC cells (Yuan et al., 2022).

#### 3.2.1 Key microbial species modulating the liver TME: *Fusobacterium* nucleatum

*Fusobacterium nucleatum* is a Gram-negative anaerobic bacterium and a known tumor-promoting bacterium that is often abundant in CRC tissues (Tilg et al., 2018). It also plays an important role in the progression of CRLMs by driving immune suppression in the liver microenvironment.

Recent evidence from patient tissue revealed that the presence of *F. nucleatum* in CRLM is associated with a reduced density of CD8^+^ T cells and an increased density of myeloid-derived suppressor cells (MDSCs), suggesting that *F. nucleatum* may contribute to immune suppression within the liver (Sakamoto et al., 2021). In response to *F. nucleatum* exposure, mice exhibit elevated plasma levels of pro-inflammatory cytokines such as IL-6 and TNF-α, along with a marked reduction in the hepatic infiltration of natural killer (NK) and T-helper 17 (Th17) cells—immune populations crucial for combating metastasis. Concurrently, an accumulation of regulatory T cells (Tregs) in the liver further fosters an immunosuppressive environment, providing a niche that supports CRC cell survival and growth (Yin et al., 2022).

Beyond direct bacterial colonization and immune modulation, *F. nucleatum* also influences the CRLM through a variety of secreted factors, one of the most intriguing being exosomes (Teng et al., 2018). These nanoscale vesicles facilitate the transfer of proteins, RNA, and lipids, which can significantly impact immune responses and tumor progression (Zhang et al., 2023). A recent study has highlighted the novel role of microbiota-associated exosomes in CRLM (Guo et al., 2020). Exosomes derived from *F. nucleatum*-infected CRC cells were found to contain miRNAs (such as miR-1246, miR-92b-3p, and miR-27a-3p) and proteins (including CXCL16, RhoA, and IL-8), which promote tumor cell migration and modulate systemic inflammation and immune responses. These exosomal markers were closely associated with *F. nucleatum* abundance and tumor stage in CRC patients, indicating their potential as biomarkers for CRLM. However, the precise mechanisms by which these exosomes modulate the liver TME remain to be fully explored.

Thus, these studies underscore *F. nucleatum* as a crucial driver of the immunosuppressive TME in CRLM, operating through both immune modulation and exosome secretion. The interplay between these mechanisms illustrates the complexity of *F. nucleatum*–host interactions, with microbial-driven metabolic and immune alterations playing a key role in CRLM progression. Disrupting these microbial influences, through targeted microbiota therapies or strategies to restore immune cell function, could enhance current treatments for metastatic CRC.

#### 3.2.2 Key microbial species modulating the liver TME: *Escherichia coli*

*Escherichia coli* is a ubiquitous bacterium that can be found in both the human gut and liver, where it has been implicated in various cancer-related processes (Arthur et al., 2012). While its role in primary CRC is largely attributed to its ability to promote tumorigenesis through the pks + genotype (Arthur et al., 2014), the pro-tumorigenic effect of *E. coli* in the liver, especially in CRLM, is distinctly driven by its inflammatory potential. In the liver, *E. coli* exerts a significant impact on immune modulation, fostering an environment conducive to metastasis.

One of the mechanisms through which *E. coli* contributes to this process is by influencing tumor metabolism (Gu et al., 2024; Wong and Yu, 2023). Lactate, as a byproduct of tumor metabolic reprogramming, has emerged as a potent factor influencing immune cell function (Xia et al., 2021). Specifically, lactate has been shown to drive M2 macrophage polarization and enhance the suppressive capabilities of regulatory T cells (Tregs), both of which contribute to an immunosuppressive TME. Furthermore, *E. coli* appears to play a pivotal role in this process, not only by altering metabolic pathways but also by influencing signaling cascades, such as the RIG-I-MAVS-NF-κB pathway, that regulate macrophage and Treg activity (Gu et al., 2024). The connection between *E. coli*-induced lactate production and immune phenotype changes challenges the traditional understanding of the microbiota as a passive factor in cancer progression. Instead, *E. coli* acts as a metabolic regulator that reprograms immune cells, creating an environment conducive to tumor growth and metastasis. The lactate-driven alteration of the immune microenvironment suggests that targeting this metabolic pathway could reprogram the immunosuppressive tumor milieu, potentially enhancing the effectiveness of current therapies. This highlights the need to consider microbiota-induced metabolic changes—specifically lactate production—as a critical factor in the development of new therapeutic strategies that target both tumor metabolism and immune modulation.

In addition to metabolic reprogramming, *E. coli* also promotes the recruitment and activation of innate immune cells, such as macrophages and inflammatory monocytes, which are key components of the hepatic pre-metastatic niche population. Translocation of *E. coli* into the liver results in preferential infiltration of these immune cells, further maintaining an inflammatory and pro-metastatic microenvironment (Bertocchi et al., 2021). In contrast, the introduction of beneficial bacteria such as *Lactobacillus paracasei* has been shown to reduce the infiltration of hepatic innate immune cells (Bertocchi et al., 2021), suggesting a protective effect against *E. coli*-induced immune reprogramming. This observation highlights the potential of microbial modulation as a therapeutic strategy to counteract the immune-dysregulatory effects of pathogenic bacteria in liver metastasis.

## 4 Microbial metabolites driving liver metastasis via gut-liver crosstalk

**FIGURE 3:**
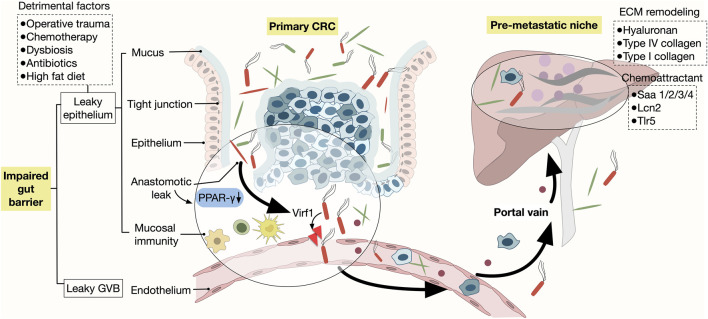
Gut microbiota-derived signals driving liver TME The influence of gut microbiota-derived metabolites on liver metastasis through the gut-liver axis is illustrated. Key interactions include microbial metabolites, such as LPS, engaging the TLR4/MD2 receptor complex, which activates the PI3K/AKT signaling pathway, facilitating CRC cell adhesion to liver sinusoidal endothelial cells. Concurrently, altered bile acid metabolism modulates NKT cell recruitment and T cell activity in the hepatic microenvironment, either promoting or suppressing metastatic progression. SCFAs, produced by commensal bacteria, can influence Th17 cell differentiation and enhance immune responses.

Microbiota-derived metabolites represent another powerful mechanism through which the gut microbiome influences liver function and pathology via gut-liver axis (Figure 3). The key metabolites—SCFAs and bile acids—are produced during microbial fermentation and metabolism within the gut (Koh et al., 2016; Lee et al., 2022). As signaling molecules, they not only regulate various physiological processes locally in the gut, but also crosstalk with the liver once released into the bloodstream. SCFAs, such as acetate, propionate, and butyrate, are produced by gut bacteria from dietary components, influencing host metabolism, epithelial barrier function, immune responses, and also liver processes like lipogenesis and gluconeogenesis (Martin-Gallausiaux et al., 2021). Bile acids, synthesized in the liver from cholesterol, are released into the small intestine as primary bile acids, where they aid in digestion and fat absorption. These primary bile acids are then modified by gut microbiota into secondary bile acids, reabsorbed in the terminal ileum and returned to the liver through the enterohepatic circulation, maintaining a balanced pool of bile acids for continuous use (Thakare et al., 2018). The gut microbiota significantly influences this bile acid metabolism, impacting not only digestive functions but also metabolic and immune regulation, thereby establishing a key link in the gut-liver axis (Guo et al., 2016; Hang et al., 2019; Campbell et al., 2020).

### 4.1 Bile acid

The pivotal role of gut microbiota in modulating liver tumor immunity through bile acid metabolism has been highlighted in a non-colorectal mouse model of liver metastases (Ma et al., 2018). This research demonstrated that *Clostridium* species can influence the growth of liver metastases by regulating the recruitment of NKT cells, a key immune cell subset involved in tumor surveillance. Specifically, the gut microbiota’s effect on bile acid metabolism has been found to play a central role in this process. Primary bile acids stimulate the expression of the chemokine CXCL16 in liver sinusoidal endothelial cells, which in turn recruits NKT cells to the liver. In contrast, secondary bile acids, produced by gut bacteria, suppress this effect. Notably, similar bile acid-mediated immune modulation patterns have been observed in human liver tissues, suggesting that this mechanism is relevant to CRLM and may open new therapeutic avenues for manipulating the gut microbiome in CRLM treatment (Dart, 2018).

In addition to NKT cells, bile acids have also been shown to affect tumor-specific CD8^+^ T cells in the liver, as evidenced in a recent study on mouse models of HCC (Varanasi et al., 2025). Elevated levels of the bile acid–conjugating enzyme BAAT (bile acid-CoA:amino acid N-acyltransferase) were found in HCC patients, with its deletion in mice leading to enhanced tumor-specific T cell responses and reduced tumor growth. Further mechanistic investigations revealed that primary bile acids induce oxidative stress in T cells, while certain secondary bile acids, such as lithocholic acid, impair T cell function through endoplasmic reticulum stress. Interestingly, ursodeoxycholic acid, a specific secondary bile acid, showed the opposite effect of lithocholic acid. Dietary supplementation with ursodeoxycholic acid has been found to mitigate this effect, restoring T cell function and enhancing the effectiveness of immunotherapy. Thus, bile acids exhibit a context-dependent and somewhat ambiguous role in regulating immune responses. While some bile acids, such as primary bile acids, impair T cell function, others, like ursodeoxycholic acid, demonstrate protective effects. This highlights the need for further research to better understand how bile acids modulate immune responses under different conditions, and how these effects can be harnessed to improve the efficacy of cancer immunotherapy.

Taken together, these findings highlight the complex dual role of bile acid metabolism in regulating liver immunity. Although current findings were primarily derived from HCC models, these insights are also highly relevant to CRLM, where similar immune suppression mechanisms in the liver TME could impair the effectiveness of immunotherapy. The modulation of bile acid metabolism could, therefore, serve as a promising therapeutic approach for improving immune responses and treatment outcomes in CRLM.

### 4.2 Short-chain fatty acids

Conversely, SCFAs are closely linked to anti-tumor effects in CRC and CRLM (Wang et al., 2019). Dysbiosis, often observed in conditions like colitis and colitis-associated CRC, leads to a reduced abundance of SCFA-producing bacteria and a subsequent decrease in SCFA levels. This imbalance impairs the metabolic and immune responses of the host (Ibrahim et al., 2019). Notably, dietary interventions, such as high-fiber diets, have been shown to shift the microbiota composition towards SCFA-producing bacteria, improving gut health and potentially enhancing anti-tumor immunity (Bishehsari et al., 2018). In mouse models of CRLM, SCFA exposure has been demonstrated to increase the abundance of beneficial probiotic bacteria, improve anti-tumor responses, and reduce liver metastasis (Ma et al., 2020). Furthermore, the role of SCFAs in the gut-liver axis has been elucidated in HCC models (Li et al., 2016). Shotgun metagenome sequencing revealed crosstalk between gut microbial metabolites, T-cell differentiation, the secretion of inflammatory cytokines, and HCC tumorigenesis. Probiotic treatment, through the production of anti-inflammatory metabolites like SCFAs, was found to reduce the migration of Th17 cells from the intestine and peripheral blood to the liver and their polarization. This modulation of immune cell trafficking helps regulate the proinflammatory immune cell population, thereby slowing tumor progression and highlighting the potential of SCFAs and probiotics in cancer therapy (Li et al., 2016).

### 4.3 Lipopolysaccharide

Lipopolysaccharide (LPS), a component of the outer membrane of Gram-negative bacteria, has been implicated in promoting tumorigenesis, with toll-like receptor 4 (TLR4) serving as its primary receptor on tumor cells (Park et al., 2009). Several studies in mouse models have demonstrated that LPS plays a critical role in promoting CRLM. A mechanistic example of this process is that LPS could enhance the metastatic capacity of CRC cells by facilitating their adhesion to the hepatic microvascular endothelial cells through the TLR4/MD2 complex, activating downstream signaling pathways such as PI3K/AKT (Hsu et al., 2011). In addition to directly influencing tumor cell behavior, LPS also shapes the immune microenvironment, contributing to an immunosuppressive milieu that hampers anti-tumor responses. Specifically, LPS has been associated with reduced efficacy of anti-PD-L1 immunotherapy in CRC, where blocking LPS or its receptor, TLR4, enhances T-cell infiltration into CRC, improves immune responses, and reduces liver metastasis (Song et al., 2018). Moreover, elevated LPS result from gut microbiota imbalance has been implicated in the secretion of cathepsin K (CTSK), which further promotes CRC metastasis by stimulating M2 polarization of tumor-associated macrophages (TAMs) through the TLR4/mTOR pathway (Li R. et al., 2019). Thus, blocking LPS within the gut-liver axis could provide a novel strategy for reducing CRC metastasis and enhancing immunotherapy outcomes.

### 4.4 Indole derivatives

Microbial metabolism generates various indole derivatives from tryptophan, which play critical roles in regulating host health and may influence cancer progression (Yu et al., 2024). These indole compounds, including I3C, IAld, and ILA, have been shown to inhibit the growth of CRC cells by reducing cell viability, promoting apoptosis (Megna et al., 2016; Sugimura et al., 2021). In addition, indole derivatives strengthen the intestinal barrier by modulating tight junctions and decreasing epithelial permeability, potentially preventing the metastatic spread of CRC to the liver via the gut-liver axis (Shin et al., 2020; Scott et al., 2020; Li et al., 2021; Jing et al., 2021). Furthermore, indoles exhibit anti-inflammatory effects by influencing immune responses, suggesting their potential to reduce chronic inflammation and inhibit CRLM (Cervantes-Barragan et al., 2017; Lei et al., 2020; Alexeev et al., 2021). However, current research remains limited, and direct evidence linking indole derivatives to CRLM is lacking. Future studies should focus on investigating the impact of indoles on CRC metastasis, particularly in the context of the gut-liver axis, to further assess their therapeutic potential in preventing or treating CRLM.

## 5 Clinical implications: focusing on immunotherapy efficacy enhancement

Among the mechanisms discussed, the influence of microbiota on immune response modulation stands out as a particularly promising strategy to overcome resistance and optimize immunotherapy. This underscores a critical opportunity for advancing the treatment of CRLM. The following section explores how the gut-liver axis can be leveraged to modulate the immune TME and enhance immunotherapy efficacy.

### 5.1 Liver as the immunologically “cold” organ

Immunotherapy, particularly immune checkpoint inhibitors (ICIs), has revolutionized cancer treatment by harnessing the immune system to combat malignancies (Chen and Mellman, 2017). The approval of ICIs, including anti-CTLA-4, anti-PD-1, and anti-PD-L1 antibodies, has led to durable clinical responses and long-term remissions in various cancers, including CRC (Topalian et al., 2012). These therapies function by blocking immune checkpoints, thereby reactivating the immune system’s ability to recognize and destroy tumor cells. However, the efficacy of ICB therapy in CRC remains limited, as only a subset of patients with microsatellite instability (MSI) or mismatch repair deficiency (dMMR) respond favorably to these treatments (Overman et al., 2018; Casak et al., 2021). The majority of CRC patients, who have microsatellite stability (MSS), do not benefit from ICB therapy, highlighting the challenge of identifying potential responders and the need to enhance the effectiveness of ICB therapy in this cohort.

Liver metastasis plays a significant role in the resistance to immunotherapy in mCRC. The liver, as an immune-tolerant organ, creates a unique microenvironment that impedes the effectiveness of ICIs. Recent clinical trials have shown that liver metastases are associated with poorer clinical outcomes in CRC patients receiving immunotherapy, including reduced survival and lower response rates (Cohen et al., 2024; Chen et al., 2023). The immune tolerance mechanisms of the liver, which include T cell anergy and suppression by myeloid-derived cells such as Kupffer cells and myeloid-derived suppressor cells, contribute to this resistance (Crispe, 2014). Moreover, liver metastasis not only disrupts local immune responses but also exerts a systemic impact on the immune system. This is evidenced by reduced T cell diversity and functionality in both patients and preclinical models with liver metastasis (Yu et al., 2021; Lindblad and Lujambio, 2021). The underlying mechanism involves the depletion of antigen-specific CD8^+^ T cells from the peripheral circulation, coupled with the induction of T cell apoptosis via Fas-FasL signaling in interaction with hepatic macrophages. This process results in the establishment of a systemic immune desert, further compromising the overall immune response and highlighting liver metastasis as a pivotal factor in immunotherapy failure in mCRC (Yu et al., 2021).

### 5.2 Harnessing the microbiota-based strategies to improve immunotherapy of CRLM

The gut microbiota, through its influence on the gut-liver axis, plays a pivotal role in modulating liver antitumor immunity. Recent studies suggest that interventions aimed at modulating the gut microbiome, such as the use of probiotics, prebiotics, or fecal microbiota transplantation (FMT), may enhance the sensitivity of CRLM to immunotherapy. These interventions can restore the balance of gut microbiota, enhance T cell priming, and improve systemic immune responses, thus mitigating the immune suppression in the liver microenvironment and promoting better outcomes in CRLM patients undergoing ICIs. Given the interplay between gut microbiota and liver immunity, targeting the gut-liver axis represents a promising strategy for overcoming the resistance to immunotherapy in mCRC and improving patient responses to treatment.

#### 5.2.1 Faecal microbiota transplantation

FMT has gained attention as a strategy to modulate the gut microbiome for improving cancer immunotherapy. By transferring microbiota from ICI-responsive donors to germ-free animals, FMT can mimic the human immune response to immunotherapy (Matson et al., 2018; Shaikh et al., 2022; Huang et al., 2022; Goc et al., 2021). In melanoma, clinical trials have shown that FMT from ICI-responsive patients can overcome resistance to anti-PD-1 therapy, highlighting its potential to enhance immunotherapy efficacy (Baruch et al., 2021; Davar et al., 2021).

Recently, pre-clinical research has demonstrated the potential of FMT as a strategy to enhance the effectiveness of immunotherapy in CRLM (Jiang et al., 2023). In patients with CRLM who did not respond to anti-PD-1 therapy, a higher abundance of *F. nucleatum* and elevated succinic acid levels were linked to immunotherapy resistance. Remarkably, fecal microbiota transfer from patients who responded well to immunotherapy—characterized by low *F. nucleatum* levels—was able to restore sensitivity to anti-PD-1 monoclonal antibody therapy in mice. This finding highlights FMT as a promising approach for reprogramming the gut microbiota and potentially overcoming resistance to immunotherapy.

#### 5.2.2 Antibiotics

Antibiotics have long been thought to impair the efficacy of ICIs, largely due to their disruptive effects on the gut microbiota. The depletion of beneficial microbial species through antibiotic treatment has been shown to diminish immune system function, resulting in impaired responses to immunotherapy (Vetizou et al., 2015; Routy et al., 2018; Gopalakrishnan et al., 2018; Xu et al., 2020). Clinical evidence supports this notion, with studies linking antibiotic use in cancer patients to poorer outcomes when receiving ICIs (Matson et al., 2018; Gopalakrishnan et al., 2018). The disruption of the microbiome seems to hinder the ability of the immune system to effectively target and destroy tumor cells, underlining the importance of a balanced gut microbiota in optimizing immunotherapy efficacy.

However, contrasting findings suggest that antibiotics might not always hinder, but at times could even enhance, the effectiveness of cancer immunotherapy, particularly by modifying the tumor microenvironment. For example, in models of CRLM, broad-spectrum antibiotics (antibiotic cocktail including vancomycin hydrochloride, neomycin sulfate and ampicillin sodium) have been observed to reduce bacterial load both in the gut and liver, leading to decreased immune suppression in the liver metastatic niche. By alleviating the immunosuppressive effects of gut-derived *E. coli*, antibiotic treatment may improve the ability of immune system to combat tumor growth, thereby enhancing the response to ICIs (Bertocchi et al., 2021).

In this context, a Phase II clinical trial investigating the combination of tadalafil, nivolumab, and oral vancomycin in HCC and CRLM patients further complicates the narrative (D'Annibale et al., 2024). Building on the observation that secondary bile acids produced by gut bacteria can suppress the recruitment of NKT cells, the trial aimed to modulate the gut microbiome in order to enhance immune responses through bile acid metabolism and improved NKT cell recruitment (Dart, 2018). However, the treatment failed to produce meaningful clinical responses. While changes in bile acid levels and immune cell activity were observed, NKT cell activity was not enhanced, likely due to the accumulation of MDSCs in the tumor microenvironment (D'Annibale et al., 2024).

Alternatively, bacteriophages provide a more targeted approach compared to antibiotics, as they can selectively eliminate specific bacterial species or strains (Shkoporov et al., 2022). This precision could help preserve beneficial microbes while addressing harmful bacteria, offering a promising alternative for enhancing cancer immunotherapy outcomes.

#### 5.2.3 Probiotics and engineered bacterial products

Probiotics and engineered bacterial products have shown promising potential in enhancing the efficacy of ICIs in CRC, renal cell carcinoma, and melanoma. Specific strains, such as *Akkermansia muciniphila*, *B. fragilis*, and *Bifidobacterium*, as well as probiotic cocktails like Probio-M9 and VB800, have demonstrated the ability to potentiate ICI activity in preclinical models (Matson et al., 2018; Vetizou et al., 2015; Routy et al., 2018; Si et al., 2022; Li Y. et al., 2019; Zhang et al., 2022; Gao et al., 2021), with VB800 currently undergoing clinical trials in combination with nivolumab for MSS CRC patients (Tanoue et al., 2019). Furthermore, bioengineered bacterial therapies are offering new solutions by directly influencing the tumor environment. For example, in CRC mouse models, genetically modified *E. coli* that enhances l-arginine availability within tumors has been shown to strengthen T-cell activity, while engineered *Lactobacillus lactis* expressing specific immune-stimulating SagA proteins could enhance the response to ICIs (Griffin et al., 2021; Canale et al., 2021).

Engineered probiotics offer a novel strategy to address the immunologically suppressed microenvironment of MSS CRLM by leveraging their natural ability to selectively colonize tumor tissue (Danino et al., 2015). For example, *E. coli Nissle 1917* has been developed as a delivery system for ICIs targeting PD-L1 and CTLA-4 (Gurbatri et al., 2020). Using a stabilized lysing release mechanism, this engineered probiotic can enable precise intratumoral delivery of nanobodies, enhancing local immune activation while reducing systemic toxicity. In preclinical models, the system outperformed traditional antibody therapies, inducing tumor regression, enhancing T-cell activation, and promoting systemic effects such as increased T-cell memory and an abscopal response. Combining this platform with cytokines like GM-CSF further amplified therapeutic efficacy, particularly in poorly immunogenic tumor models (Gurbatri et al., 2020). This innovative approach integrates synthetic biology with immunotherapy to refine checkpoint blockade delivery and improve outcomes in MSS CRLM.

Similarly, an innovative LPS-trap nanoparticle system has shown substantial potential in enhancing immunotherapy for CRLM. By selectively targeting and neutralizing LPS within the tumor microenvironment, this system could mitigate LPS-driven immunosuppression, restore T-cell infiltration, and significantly improve anti-PD-L1 therapy outcomes. In mouse models of MSS CRC, the LPS-trap system not only boosted immunotherapy efficacy but also effectively reduced liver metastases, highlighting its transformative role in modulating the gut-liver axis to enhance therapeutic outcomes (Song et al., 2018).

Recently, *F. nucleatum*, traditionally regarded as a pathogenic bacterium in CRC, has been revealed to play a paradoxical role in enhancing immunotherapy efficacy in MSS CRC (Wang et al., 2024). Specifically, research indicated that intratumoral *F. nucleatum* can produce butyric acid, which modulated CD8^+^ T cells by inhibiting histone deacetylase activity. This inhibition was shown to reduce PD-1 expression and reactivate T cell function, alleviating immune exhaustion and improving therapeutic responses. Both clinical and preclinical data further demonstrated that elevated levels of *F. nucleatum* within tumors were associated with improved outcomes in MSS CRC patients treated with anti-PD-1 therapy. These findings suggest a dual role for *F. nucleatum*—as both a pathogen and a potential enhancer of immunotherapy—emphasizing the intricate and multifaceted relationship between tumor-associated microbiota and immune modulation.

Given the contradictory findings and the complexity of underlying mechanisms, combining microbiota-based approaches with ICI therapy requires careful consideration. Future research should focus on the design and validation of targeted microbiota therapies to ensure both safety and efficacy, with their potential thoroughly evaluated in well-designed clinical trials.

#### 5.2.4 Clinical relevance

As discussed above, microbiota-based interventions, including FMT, probiotics, prebiotics, and engineered bacteriophages, hold significant potential in CRC treatment by enhancing chemotherapy, improving immunotherapy efficacy, and mitigating treatment-associated side effects (Qu et al., 2023; Wang et al., 2023; White and Sears, 2024; Lang et al., 2023). While some of these strategies remain in preclinical stages, others have advanced to clinical trials, highlighting their translational relevance. A detailed summary of key clinical trials exploring microbiota applications in CRC is presented in (Table 1).

**TABLE 1:** A detailed summary of key clinical trials exploring microbiota applications in CRC.

Clinical trial	Intervention	Cancer therapy	Patients	Phase	Primary outcome	Status
Anti-tumor therapy						
NCT05279677	FMT	Immune checkpoint inhibitor and TKI	CRC patients with advanced stage	Phase II	Objective response rate	Recruiting
NCT04729322	FMT	Re-introduction of anti-PD-1 therapy (Pembrolizumab or Nivolumab)	mCRC patients not responding to anti-PD-1	Phase II	Objective response rate	Active, not recruiting
NCT04131803	Probiotics	Standard chemotherapy plus targeted therapy	Unresectable mCRC	Unknown	Objective response rate	Recruiting
NCT04208958	VE800	Nivolumab	Selected types of advanced or metastatic cancer	Phase I/II	Safety and tolerability of VE800 in combination with nivolumab	Completed
NCT04682665	Eicosapentaenoic Acid	—	CRLM	Observational	Abundance of individual bacterial taxa in the gut microbiome	Completed
NCT03428477	Icosapent Ethyl	CRLM surgery	Planned liver resection surgery for CRLM	Phase III	Progression free survival	Active, not recruiting
NCT06728072	Microbiome modulation Therapy	Chemotherapy	Stage IV CRC	Phase II	Best response by analysis using response evaluation criteria in solid tumors	Not yet recruiting
NCT06349590	Standardized preoperative diet	Surgery	CRC	Phase I/II	Change in lab values of the dietary intervention on the collagenolytic potential of the microenvironment	Recruiting
NCT03785210	Oral Vancomycin	Nivolumab and Tadalafil	Refractory primary HCC or liver dominant metastatic cancer from colorectal or pancreatic cancers	Phase II	Best overall response	
Tumor supportive care						
NCT05570942	Oral *Lactobacillus* Rhamnosus TCELL-1	Chemotherapy	Stage III CRC	Phase II	Changes in quality of life	Unknown status
NCT03705442	Probiotics	Cancer therapy	mCRC	Phase II	Incidence of grade III/IV diarrhea	Unknown status
NCT03782428	Probiotic	Surgery	CRC	Unknown	Level of circulating inflammatory cytokines (TNF-α, IFN-γ, IL-6, IL-10, IL-12, IL-17A, IL17C, IL-22) pre and post intervention	Completed
NCT01895530	Probiotic	Surgery	CRC	Unknown	Difference in gene expression of cytokines	Completed
NCT06561516	Red Ginseng	Surgery	Gastrointestinal cancer	Unknown	Changes in microbiome composition based on the consumption or non-consumption of red ginseng following gastrointestinal cancer surgery	Completed
NCT01916239	Pomegranate extract	Surgery	CRC	Phase I/II	1. Phenolics and derived metabolites in colon tissues, plasma and urine 2. Gene expression profiling in colon tissues	Completed
NCT00936572	Probiotic	Surgery	CRC	Phase II	Morphological and microbiological evaluation of the colonic microflora, gastrointestinal function	Completed
NCT04821258	Nutraceutical “MICODIGEST 2.0″	Surgery	CRC	Unknown	Rate of complications	Unknown status
NCT04869956	Dietary Intervention	Surgery	CRC	Unknown	1. Rate of anastomotic leakage 2. Surgical site infection	Unknown status
NCT05039060	Modified MAC Diet	Surgery	CRC	Unknown	The change of gut microbiota diversity	Unknown status
NCT02169388	Chemotherapy	Chemotherapy	CRC	Phase I	1. Composition of microorganisms in stool after probiotic intervention 2. SCFAs in feces of patients after chemotherapy 3. Frequency and severity of adverse effects during chemotherapy	Unknown status
NCT06202183	Exercise	Chemotherapy	Early-stage or metastatic CRC	Unknown	Gut microbiome genomes	Recruiting
NCT01410955	Probiotic	Chemotherapy	CRC patients started new line of chemotherapy based on irinotecan	Phase III	Prevention of grade 3–4 diarrhea by probiotics in patients treated by irinotecan-based chemotherapy	Completed
NCT00197873	*Lactobacillus* Rhamnosus	Chemotherapy-related diarrhea	mCRC	Unknown	Effect on the treatment-related grade 2 to 4 diarrhea	Completed
NCT06456229	Perioperative Probiotics	Surgery	CRC	Unknown	Time to bowel movement	Recruiting

FMT, fecal microbiota transplantation; CRC, colorectal cancer; TKI, tyrosine kinase inhibitor; mCRC, metastatic colorectal cancer; Hepatocellular Carcinoma, HCC; PD-1, Programmed Cell Death Protein 1; CRLM, colorectal liver metastasis; TNF-α, tumor necrosis factor alpha; IFN-γ, interferon gamma; IL-6, Interleukin 6; IL-10, Interleukin 10; IL-12, Interleukin 12; IL-17A, Interleukin 17A; IL-17C, Interleukin 17C; IL-22, Interleukin 22; MAC, modified atkins diet; SCFAs, Short-Chain Fatty Acids.

Microbiota-enhanced immunotherapy has shown promising results in clinical trials involving renal cell carcinoma (RCC) and melanoma (Baruch et al., 2021; Davar et al., 2021; Dizman et al., 2022). In CRC, preclinical evidence, particularly from murine studies, further supports the potential of microbiota-modulated immunotherapy. For example, a study assessing the combined effect of FMT and anti-PD-1 therapy in colorectal tumor-bearing mice demonstrated significantly improved survival and tumor control when compared to either treatment alone. Metagenomic analyses revealed notable shifts in the gut microbiota composition, with specific genera, including *Bacteroides*, identified as crucial contributors to the enhanced efficacy of anti-PD-1 therapy (Huang et al., 2022). Furthermore, metabolomic analyses identified potential metabolites, such as punicic acid and aspirin, which may exert immunomodulatory effects that further support the therapeutic impact of immunotherapy (Xu et al., 2020). These findings have deepened our understanding of how microbiota interventions can improve the clinical effectiveness of PD-1 inhibitors in CRC.

Clinical trials investigating microbiota-based interventions in conjunction with immunotherapy have also demonstrated encouraging results. A recent phase Ib/II trial exploring the combination of regorafenib and toripalimab in metastatic CRC reported an objective response rate (ORR) of 15.2% and a disease control rate (DCR) of 36.4%. The study found that *Fusobacterium* abundance in baseline fecal samples was associated with poor progression-free survival (PFS), suggesting that gut microbiota composition may influence treatment outcomes (Wang et al., 2021). Furthermore, a phase II trial evaluating the combination of FMT, tislelizumab, and fruquintinib in refractory MSS metastatic CRC revealed improved survival, with a median PFS of 9.6 months and an ORR of 20%. Notably, patients with high abundance of *Proteobacteria* and Lachnospiraceae showed better treatment response (Zhao et al., 2023). In a separate study (NCT04208958), the combination of VE800 with nivolumab in metastatic CRC demonstrated a median overall survival of 7.6 months and a PFS of 1.8 months. Although these results were limited, they highlight the potential of microbiota interventions to enhance immune checkpoint blockade. Together, these studies underscore the growing potential of microbiota modulation as a complementary strategy to enhance the efficacy of immunotherapy in CRC, providing promising therapeutic options for patients with refractory disease.

## 6 Discussion and perspective

### 6.1 Complex and context-dependent role of microbiota in CRLM

The process of CRLM follows a multi-step cascade, where microbiota and their metabolites influence each stage, from local invasion of CRC cells to their colonization and growth in the liver. This cascade is critically regulated through the gut-liver axis, a bidirectional communication pathway between the gut microbiota and the liver. The role of microbiota in each step of this cascade is complex and, in many cases, contradictory. While some microbes and microbial products promote cancer progression, others can enhance immune responses or mitigate tumor growth. As such, understanding the role of microbiota in the CRLM cascade is crucial for uncovering therapeutic strategies that can manipulate the gut-liver axis to improve immunotherapy outcomes in liver metastasis.

The influence of microbiota begins in the primary tumor, where dysbiosis plays a pivotal role in CRC progression and invasion. Certain bacterial species, such as *F. nucleatum*, *E. coli*, and *B. fragilis* contribute to tumorigenesis by creating a pro-inflammatory microenvironment and altering immune cell activity (Tilg et al., 2018; Gao et al., 2022; Sears and Garrett, 2014). These microorganisms have been shown to modulate the immune response in ways that promote tumor invasion and progression. However, the role of theses microbiota is not purely negative. In fact, some of them have been identified to enhance the efficacy of immunotherapy under certain conditions (Yuan et al., 2022; Wang et al., 2024), complicating the overall understanding of microbiota as both tumorigenic and immune-modulating agents.

Similarly, antibiotics, which are commonly used in the treatment of cancer patients to prevent infections, have a complex and often contradictory effect on immunotherapy outcomes. On the one hand, antibiotics can impair the gut microbiota, reducing diversity and disrupting the balance of beneficial microbes that support immune function. This disruption is often associated with reduced efficacy of ICB therapies, as the microbiota is crucial for the optimal activation of immune responses (Vetizou et al., 2015; Routy et al., 2018; Gopalakrishnan et al., 2018; Xu et al., 2020). On the other hand, the use of antibiotics may also reduce the abundance of certain pathogenic microbes that actively suppress the immune system. Thus, while antibiotics can inadvertently reduce the immune-enhancing potential of the microbiota, they may also provide benefits by alleviating microbial-induced immunosuppression (Bertocchi et al., 2021; Dart, 2018). The challenge lies in identifying the specific microbial species and understanding the precise mechanisms by which antibiotics influence immunotherapy efficacy, as the effects can vary depending on the type of bacteria being targeted.

Bile acids, a class of metabolites produced by the liver and influenced by gut microbiota, further exemplify the dual role of microbiota in immune modulation. Primary bile acids, synthesized in the liver, play a key role in regulating immune responses in the liver, particularly through the recruitment of NKT cells, which are essential for anti-tumor immunity (Dart, 2018). However, certain secondary bile acids, such as lithocholic acid, impair the function of tumor-specific T cells by inducing endoplasmic reticulum stress (Varanasi et al., 2025). To add to the complexity, ursodeoxycholic acid, another secondary bile acid was shown to enhance T cell function and improve the response to immunotherapy (Varanasi et al., 2025). This highlights the complex and often contradictory effects of bile acids in immune modulation and suggests that manipulating bile acid metabolism could offer a promising therapeutic strategy to enhance immunotherapy efficacy in CRLM.

### 6.2 Future directions in gut microbiome and immunotherapy research

To resolve the contradictions in the dual roles of microbiota and optimize its therapeutic potential, future research should focus on developing targeted microbiota-based interventions that selectively enhance beneficial microbes while suppressing harmful ones. Personalized microbiome modulation tailored to an individual’s microbial composition, could maximize immune-boosting effects and minimize tumor-promoting influences (Ratiner et al., 2024). Advances in metagenomic sequencing and microbiome analysis will be key to identifying specific microbial populations and their metabolites that influence CRC progression and immunotherapy response (Gu et al., 2019). Additionally, a deeper understanding of the gut-liver axis, particularly how microbiota and metabolites such as bile acids affect liver immunity, will provide new targets for improving CRLM treatment. The use of bacteriophages, capable of selectively eliminating pathogenic bacteria, offers a promising approach for achieving high specificity in microbiota modulation without disturbing beneficial microbes (Shkoporov et al., 2022). Combining these insights with current cancer therapies may lead to more effective, personalized treatment strategies for CRC and CRLM patients.

Future research on the gut microbiome in cancer immunotherapy should focus on larger clinical trials to better understand its role in treatment outcomes. Current studies with limited sample sizes need to be expanded to facilitate more robust subgroup analyses, accounting for patient health status, cancer types, and immunotherapy regimens (Sharafi et al., 2022). This approach will help clarify how the microbiome impacts responses to treatments, as seen in the varying relevance of PD-L1 expression in different cancer subtypes. Furthermore, precise classification of patient responses using criteria like response evaluation criteria in solid tumors (RECIST) will provide more detailed insights into the microbiome’s influence on treatment efficacy.

In addition, combination immunotherapies are becoming more common, but their impact on immune-related adverse events (irAEs) and treatment outcomes, particularly in relation to the gut microbiome, remains underexplored (Baxi et al., 2018). Exploring biomarkers for irAEs in these settings is crucial. Advances in metagenomics and multi-omics approaches offer promising tools to uncover the microbiome’s functional role, which could inform more targeted therapies. New technologies such as microbiome imaging and artificial intelligence-based models could enhance predictive capabilities, while standardizing methods across studies for immunotherapy trials will ensure more reliable results.
